# A Comprehensive Study of Synthesis and Analysis of Anisotropic Iron Oxide and Oxyhydroxide Nanoparticles

**DOI:** 10.3390/nano12234321

**Published:** 2022-12-05

**Authors:** Elizaveta Chernova, Vladimir Botvin, Maria Galstenkova, Yulia Mukhortova, Dmitry Wagner, Evgeny Gerasimov, Maria Surmeneva, Andrei Kholkin, Roman Surmenev

**Affiliations:** 1 International Research & Development Center Piezo and Magnetoelectric Materials, Research School of Chemistry & Applied Biomedical Sciences, National Research Tomsk Polytechnic University, 634050 Tomsk, Russia; 2 Physical Materials Science and Composite Materials Centre, Research School of Chemistry & Applied Biomedical Sciences, National Research Tomsk Polytechnic University, 634050 Tomsk, Russia; 3 Scientific Laboratory for Terahertz Research, National Research Tomsk State University, 634050 Tomsk, Russia; 4 Department of Catalyst Research, Boreskov Institute of Catalysis SB RAS, 630090 Novosibirsk, Russia; 5 School of Natural Sciences and Mathematics, Ural Federal University, 620000 Ekaterinburg, Russia

**Keywords:** iron oxides, magnetite, anisotropic nanoparticles, co-precipitation, methodological scheme

## Abstract

One-dimensional anisotropic nanoparticles are of great research interest across a wide range of biomedical applications due to their specific physicochemical and magnetic properties in comparison with isotropic magnetic nanoparticles. In this work, the formation of iron oxides and oxyhydroxide anisotropic nanoparticles (ANPs) obtained by the co-precipitation method in the presence of urea was studied. Reaction pathways of iron oxide and oxyhydroxide ANPs formation are described based on of X-ray diffraction (XRD), Raman spectroscopy, X-ray photoelectron spectroscopy (XPS), scanning electron microscopy (SEM), high-resolution transmission electron microscopy (HRTEM), and pulse magnetometry studies. It is shown that a nonmonotonic change in the Fe_3_O_4_ content occurs during synthesis. The maximum content of the Fe_3_O_4_ phase of 47.4% was obtained at 12 h of the synthesis. At the same time, the reaction products contain ANPs of α-FeOOH and submicron isotropic particles of Fe_3_O_4_, the latter formation can occur due to the oxidation of Fe^2+^ ions by air-oxygen and Ostwald ripening processes. A subsequent increase in the synthesis time leads to the predominant formation of an α-FeOOH phase due to the oxidation of Fe_3_O_4_. As a result of the work, a methodological scheme for the analysis of iron oxide and oxyhydroxide ANPs was developed.

## 1. Introduction

Recently, one-dimensional (1D) nanosized structures (nanorods, nanowires, nanoellipsoids, nanoneedles) including iron oxides and oxyhydroxides have been in the focus of scientific research due to their fundamental importance and practical significance for materials science and medicine [1,2,3,4]. For example, magnetite (Fe_3_O_4_) nanorods have found many applications in industry as magnetic storage devices, catalysts, cooling devices, gas sensors, electrodes in lithium-ion batteries, as well as in various medical diagnostics contexts [5,6]. Anisotropic nanoparticles (ANPs) of FeOOH can be used as selective sorption materials [7], catalysts [8], and templates for the synthesis of other iron oxides compounds [9]. The transition from traditional isotropic to ANPs is accompanied by a change in their optical (spectral), biological, magnetic properties, chemical reactivity, and catalytic activity [10]. This is demonstrated not only for iron-based nanoparticles, but also for other valuable nanoobjects [11,12]. Anisotropic gold nanoparticles of different aspect ratio exhibit a shift of the surface plasmon-absorption spectra further into the near-infrared region that improve in vivo tissue penetration in comparison with isotropic nanoparticles [13]. ANPs offer a higher catalytic activity than isotropic alternatives due to the more developed specific surface area and increase in the number and type of active centers [14]. The anisotropic shape of nanoparticles also affects their magnetic properties [15,16,17]. In contrast to the homogeneous magnetization of isotropic particles, anisotropic nanoparticles are easily magnetized along the longest axis (easy magnetization axis) to generate an anisotropic response to magnetic fields [3,18].

The complexity in obtaining pure iron oxides and oxyhydroxides lies in its tendency to redox reactions due to the transitional oxidation state of iron. Therefore, oxygen-containing iron compounds can be found in the reaction products in the form of several different phases and transform into each other under certain synthesis conditions [19]. The main difficulty in identifying phases of iron derivatives is due to the simultaneous presence of synthesis products having similar properties, necessitating the use of a sufficient number of the most informative research methods. Some of the iron hydroxides tend to form ANPs directly during synthesis due to the peculiarities of their crystal structure. Thus, goethite (α-FeOOH) forms needle-like particles [20], while another oxyhydroxide, akaganeite (β-FeOOH), crystallizes in the form of nanoellipsoids [21].

A number of synthesis methods have been used in the attempt to obtain anisotropic Fe_3_O_4_ nanoparticles [5,22]. The complexity of the single-stage preparation of Fe_3_O_4_ ANPs is associated with its cubic crystal structure as well as with different deposition rates of the Fe^2+^ and Fe^3+^ compounds used as initial reagents in the synthesis by salt co-precipitation [23]. In this regard, basic approaches to the synthesis of Fe_3_O_4_ nanorods can be divided into three groups: (1) single-stage methods with the use of surfactants limiting growth of crystallographic planes [24]; (2) two-stage synthesis through the formation of intermediate compounds with anisotropic particles (α- or β-FeOOH) [9,25]; (3) hydrothermal synthesis from preliminarily synthesized isotropic Fe_3_O_4_ nanoparticles [26]. Among the mentioned methods, one-stage synthesis is of particular interest due to the possibility of using it to obtain ANPs having controlled properties without the use of additional reagents. One-stage synthesis can be carried out under slow and controlled precipitation in the presence of urea that leads to gradual increase in the concentration of hydroxide anions (form under dissociation of ammonia solution) participating in formation of target Fe_3_O_4_ ANPs [27,28]. Along with the importance of the one-stage method itself, its mechanism is of undoubted interest, which takes into account the ongoing reactions and, as a result, makes it possible to obtain ANPs with certain characteristics. Despite the mentioned well-known studies discussing the features of the mechanism of nanoparticle synthesis in the presence of urea, to the best of our knowledge, not all features of the synthesis are taken into account. This makes the controlled synthesis of Fe3O4 ANPs complicated.

Along with the synthesis of ANPs, informative methods for their study are of considerable interest, especially in terms of confirming the reliability of the obtained results. Phase and functional composition of the ANPs are studied by X-Ray diffraction (XRD) [29] and Infrared spectroscopy [30,31]. The morphology and shape of ANPs is studied by scanning electron microscopy (SEM) and transmission electron microscopy (TEM), including high-resolution TEM (HRTEM) [32,33]. In the case of magnetic ANPs, saturation magnetization (M_s_) is measured to determine magnetic characteristics important for biomedical applications [34,35].

Thus, despite a fairly large number of studies, the synthesis of iron-based ANPs, having controlled size, shape, phase composition, and other properties, remains an urgent and yet-unsolved problem. Additionally, there is still no reliable set of research methods for revealing correlations between the morphology and composition of ANPs. In this regard, this work is devoted to the preparation of anisotropic iron oxides and oxyhydroxide nanoparticles and their detailed analysis in order to determine a set of analytical methods for studying the structure, composition, and physicochemical properties of individual iron oxides (oxyhydroxide) and their mixtures.

## 2. Materials and Methods

### 2.1. Materials

FeCl_3_∙6H_2_O, FeSO_4_∙7H_2_O and (NH_2_)_2_CO (urea) powders were purchased from Sigma Aldrich (Steinheim, Germany) and used without further purification. Deionized water obtained by the Millipore Milli-Q system (Darmstadt, Germany) was used in all experiments.

### 2.2. Synthesis of Anisotropic Nanoparticles

**Figure 1:**
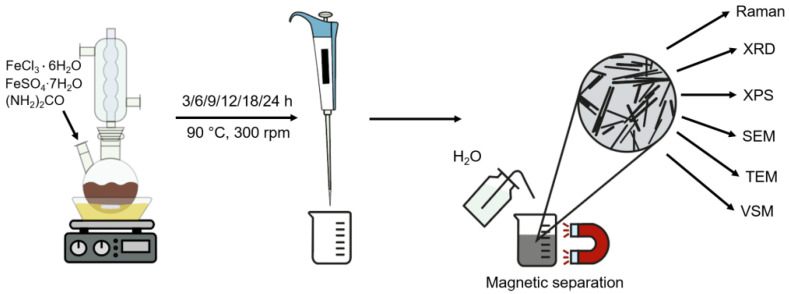
Methodology of the synthesis and investigation of iron oxide ANPs.

In order to synthesize ANPs, we used a method adapted from Lian et al. [27]. The calculated amount of FeCl_3_∙6H_2_O (6.756 g, 0.250 M), FeSO_4_∙7H_2_O (3.426 g, 0.123 M), urea (12.0 g, 2.0 M), and 100 mL of deionized water were placed into a two-necked round flask equipped with a reverse condenser (Figure 1). The obtained solution was stirred by magnetic stirrer at 90–95 °C in an oil bath (300 rpm). After 3, 6, 9, 12, 18, and 24 h of the synthesis, a 15 mL probe sample was taken for further study. The resulting particles were separated by the magnet and washed several times with deionized water to a neutral pH.

Lian et al. suggest that the formation of Fe_3_O_4_ occurs according to the following chemical reactions [25]:

(NH_2_)_2_CO + H_2_O → 2NH_3_ + CO_2_ (t > 85 °C) (1)

NH_3_ + H_2_O → NH_3_∙H_2_O → NH_4_^+^ + OH^−^ (2)

Fe^3+^ + 3OH^−^ → Fe(OH)_3_ (3)

Fe(OH)_3_ → FeOOH + H_2_O (4)

Fe^2+^ + 2OH^−^ → Fe(OH)_2_ (5)

2FeOOH + Fe(OH)_2_ → Fe_3_O_4_ + 2H_2_O (6)

### 2.3. Characterization of the Samples

The phase composition of ANPs was analyzed by XRD on a Shimadzu XRD 6000 (Shimadzu Corporation, Kyoto, Japan) diffractometer (CuKα radiation) at a range of 10 to 70° (scan rate 1°/min). The XRD data were analyzed by the Rietveld method using the Match! Software (v. 3.13, Bonn, Germany) to assess the phase composition of the samples.

Raman spectra were recorded on an NT-MDT system (NT-MDT Spectrum Instruments, Zelenograd, Russia) equipped with a 100× objective. Excitation was performed with a laser at wavelengths of 633 nm with a maximum power of 60 mW. To prevent heating and oxidation of magnetite, no more than 1% of the laser power was used.

To characterize the surface of ANPs, X-ray photoelectron spectroscopy (XPS) was performed using a Thermo Fisher Scientific XPS NEXSA spectrometer (Thermo Fisher Scientific, Waltham, MA, USA) with a monochromated Al Kα Alpha X-ray source working at 1486.6 eV. XPS survey spectra were acquired at a pass energy of 200 (eV) and energy resolution of 1 eV from the surface area of 200 µm^2^. The high-resolution spectra were acquired at a pass energy of 50 eV and energy resolution 0.1 eV. A flood gun was used to compensate for the charge.

Prior to the study of the morphology, a conductive Au coating was deposited on the samples. SEM (Quanta 200 3D electron microscope (FEI Company, Hillsboro, OR, USA)) equipped with the energy dispersive spectroscopic analysis (EDS) (FEI Company, Hillsboro, OR, USA) was performed to evaluate the changes in the morphology and elemental composition of the samples.

The structure of the samples was studied using HRTEM (ThemisZ electron microscope, Thermo Fisher Scientific, Waltham, MA, USA) with an accelerating voltage of 200 kV and a limiting resolution of 0.07 nm. The images were recorded using a Ceta 16 CCD sensor (Thermo Fisher Scientific, Waltham, MA, USA). The device is equipped with a SuperX (Thermo Fisher Scientific, USA) energy-dispersive characteristic X-ray spectrometer (EDX) with a semiconductor Si detector with an energy resolution of 128 eV.

The magnetic properties of ANPs were investigated at a temperature of 295 K with an external pulsed magnetic field up to 6.5 kOe using a pulsed magnetometer (Tomsk State University, Tomsk, Russia).

## 3. Results and Discussion

**Figure 2:**
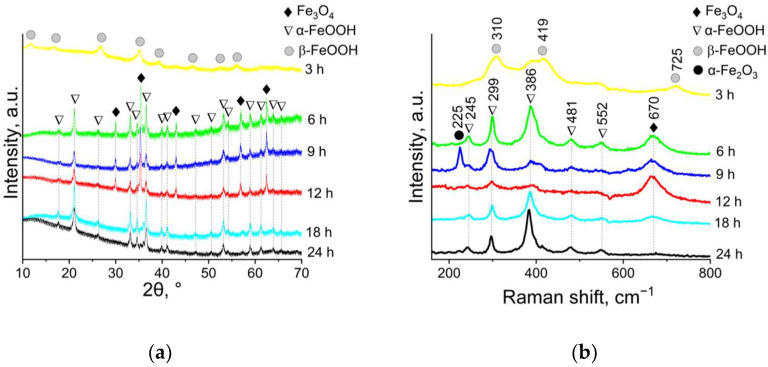
XRD patterns (**a**) and Raman spectra (**b**) of ANPs.

To study the formation of ANPs in detail and determine occurring reactions and processes, an evaluation was carried out at different synthesis times. The structure, phase and chemical composition of ANPs were studied by XRD, Raman spectroscopy, and XPS. Figure 2a shows the XRD pattern of the samples at different synthesis times. After 3 h of synthesis, XRD patterns corresponded to akaganeite (β-FeOOH) (PDF card 96-900-2991). With a further increase in the synthesis time, the XRD patterns contain characteristic reflections of Fe_3_O_4_ (PDF card 96-900-5838) at 2θ = 30.0, 35.37, 37.0, 43.00, 56.88, and 62.43, corresponding to the hkℓ planes in the crystal with (220), (311), (222), (400), (511), and (440) [36,37], as well as characteristic reflections of goethite (α-FeOOH) (PDF card 96-901-0407) at 2θ = 17.77, 21.11, 26.28, 33.13, 34.58, 36.55, 39.96, 41.16, 47.22, 50.62, 53.15, 54.14, 58.88, 61.23, 63.89, and 65.64, corresponding to the hkℓ planes in the crystal with (020), (110), (120), (130), (021), (111), (121), (140), (221), (151), (002), and (061), respectively [38]. After 12 h of synthesis, the intensity of magnetite peaks decreases and the intensity of goethite peaks increases. The quantitative phase composition of the samples is presented in Table 1. The results of Raman spectroscopy (Figure 2b) are in agreement with XRD data. The spectrum of the sample after 3 h of synthesis contains the characteristic peak of β-FeOOH at 310, 419, and 725 cm^−1^. The Raman spectra of the samples after 6, 9, 12, 18, and 24 h of synthesis include two peaks of α-FeOOH located at 299 and 386 cm^−1^, in addition to other less intense peaks at 245, 299, 386, 481, and 552 cm^−1^. An increase in the synthesis time up to 12 h leads to an intensification of the characteristic peak of Fe_3_O_4_ at 670 cm^−1^. A further increase of the time of the synthesis is accompanied by a gradual decrease of the intensity of the 670 cm^−1^ peak. A broadening of the magnetite peak is observed due to the formation of maghemite (γ-Fe_2_O_3_), which generally occurs due to the presence of Fe^2+^ in the Fe_3_O_4_ structure as a result of oxidation both by air oxygen during the synthesis and by laser beam in the process of Raman spectra registration.

**Figure 3:**
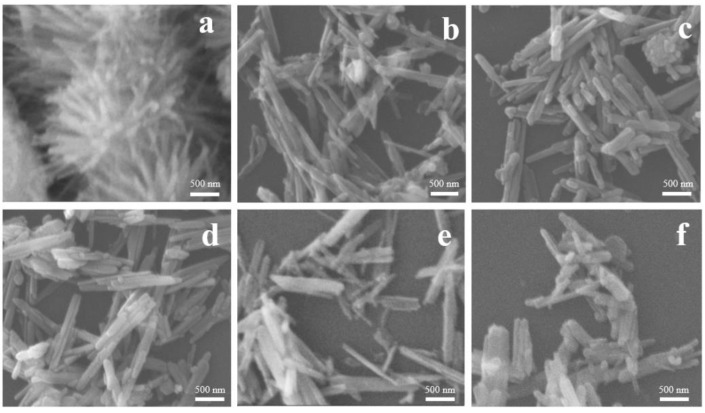
SEM microphotographs of ANPs after 3 (**a**), 6 (**b**), 9 (**c**), 12 (**d**), 18 (**e**), and 24 h (**f**) of synthesis.

The SEM images of the obtained samples presented in Figure 3 reveal details of their morphology. After 3 h of synthesis, β-FeOOH needle-like structures began to form. Increase of the synthesis time should lead to transformation of β-FeOOH needles to α-FeOOH nanorods, which interact with Fe(OH)_2_ during increasing pH of the solution (formation of ammonia via urea decomposition) according to the mechanism proposed by Lian et al. [27] to eventually form Fe_3_O_4_ nanorods. However, SEM images in combination with XRD and Raman results do not reliably confirm the formation of magnetite nanorods.

**Figure 4:**
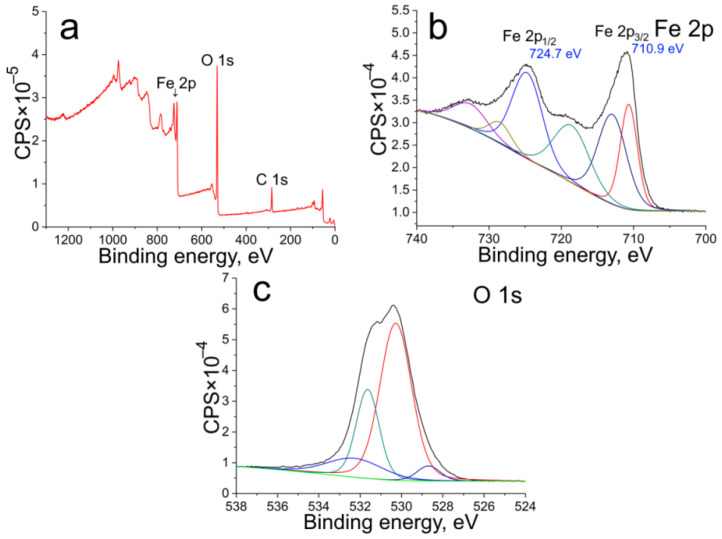
Survey (**a**), Fe 2p (**b**), and O 1s (**c**) XPS spectra of ANPs obtained during 12 h synthesis.

XPS analysis was used to more precisely reveal the chemical, phase, and molecular composition of the synthesized ANPs (Figure 4). Survey spectra demonstrate predominant peaks of Fe and O. There are also peaks of C as an adventitious carbon and peaks of S and Cl of initial iron salt at the earliest stage of the synthesis. High-resolution Fe 2p and O 1s XPS spectra in the case of all time intervals of the synthesis have similar peaks because of the close position of peaks energy of α-FeOOH and Fe_3_O_4_ as the main phases [39]. Fe 2p spectra include Fe p_3/2_ and Fe 2p_1/2_ peaks at 711 and 724 eV, respectively, which are characteristic of iron oxide and oxyhydroxide derivatives (Table 2) [40]. Deconvoluted spectra contain peaks of Fe^3+^ and Fe^2+^ presented in the surface of all studied samples. XPS O 1s spectra demonstrate 4 predominant peaks at 528.7, 530.3, 531.6, and 532.3 eV corresponding to surface OH, lattice Fe–O, lattice Fe–OH, and adsorbed H_2_O, respectively [41]. An increase in the Fe/O ratio during synthesis (until 12 h) is connected with decreased oxygen content due to the formation of Fe_3_O_4_. After 12 h of synthesis, the Fe/O ratio starts to decrease. A slight decrease in the Fe/O ratio after 12 h may be due to the formation of OH groups on the surface of the samples.

**Figure 5:**
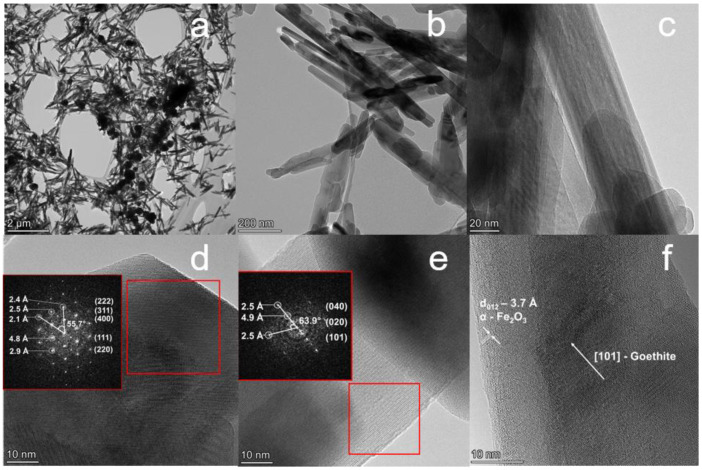
TEM micrographs of the sample obtained during the synthesis for 12 h: (**a**,**b**) survey images of synthesized nanoparticles; (**c**) HRTEM image of micro pores in ANPs of α-FeOOH; (**d**) HRTEM image of isotropic Fe_3_O_4_ particles; (**e**) HRTEM image of anisotropic α-FeOOH particles; (**f**) HRTEM image of α-Fe_2_O_3_ layer on the α-FeOOH particle (red insets show FFT-images with calculations of interplanar spacing).

For a detailed study of the morphology and fine structure of ANPs, TEM images were recorded. Figure 5 shows TEM and HRTEM images of the sample obtained during 12 h.

**Figure 6:**
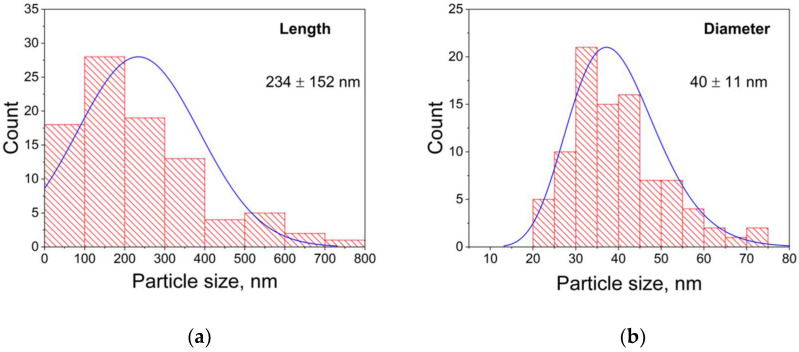
Distribution of length (**a**) and diameter (**b**) of obtained ANPs by size.

Analysis of microphotographs indicates that the sample contains mainly ANPs of various sizes (Figure 5a,b). The length and diameter distributions of the ANPs are shown in Figure 6. The sample also contains submicron particles of about 200–600 nm in size, which are agglomerates of two or more particles (Figure 5a).

In addition to the XRD and Raman spectroscopy results, the phase composition of the ANPs is described by the detailed study of HRTEM images (Figure 5d,e) using interplanar spacing (FFT analysis). Since the complexity of the analysis of iron oxides and oxyhydroxides ANPs lies in their close interplanar spacings (Table 3), the FFT analysis should simultaneously take into account several values of interplanar spacings. According to FFT analysis of submicron isotropic particles (Figure 5d), these relate to Fe_3_O_4_ to confirm interplanar spacings of 2.4, 2.5, 2.1, 4.8, and 2.9 Å corresponding to (222), (311), (400), (111), and (220) crystallographic planes, respectively. Conversely, ANPs correspond to the α-FeOOH phase that supports by the characteristic interplanar spacings of 2.5, 4.9, and 2.5 Å corresponding to (040), (020), and (101) crystallographic planes, respectively (Figure 5e). The investigated interplanar spacings in the HRTEM images are in agreement with the XRD data (Table 3). In addition, measured angles between the planes equal 55.7° and 63.9° also confirm that these particles belong to the Fe_3_O_4_ (Figure 5d) and α-FeOOH (Figure 5e) phases, respectively.

In some cases of ANPs morphology micro pores were observed (Figure 5c). They can be formed during the dehydroxylation of α-FeOOH in the process of TEM study under the influence ultra-high vacuum and the energy of the beam of the microscope [45]. This is consistent with the XRD results, since the nonuniform broadening of the diffraction peaks in the samples can be explained by the formation of pores [46]. At the same time, a layer of hematite (α-Fe_2_O_3_) of about 13.5 nm with interplanar spacing of 3.7 Å is observed on the surface of the goethite ANPs [47], which is formed as a result of its dehydroxylation and transformation to a more stable α-Fe_2_O_3_ phase (Figure 5d) [19]. These disordered crystallites have sizes within the range of 1–3 nm. Thus, the obtained after 12 h reaction mixture includes both isotropic Fe_3_O_4_ nanoparticles and ANPs of α-FeOOH.

**Figure 7:**
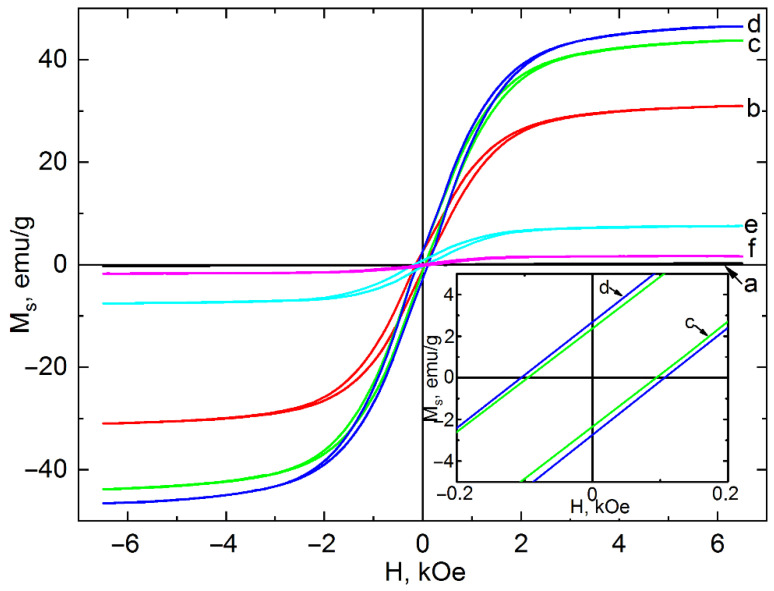
Hysteresis loops of samples after 3 (**a**), 6 (**b**), 9 (**c**), 12 (**d**), 18 (**e**), and 24 h (**f**) of synthesis.

It is known that the magnetic properties of magnetite particles can be affected by various factors, of which the most significant are the size and shape of crystallites, the presence of weakly magnetic impurities and crystallinity. The value of the Ms of magnetite decreases with a low crystallite size and with the presence of weakly magnetic impurities in the material [48,49]. The hysteresis loops of the synthesized nanoparticles Fe_3_O_4_ and their magnetic characteristics are shown in Figure 7 and in Table 4, respectively.

Synthesis for 3 h did not lead to the formation of the magnetite phase. The sample consisted of akageneite β-FeOOH, which, although paramagnetic at a temperature of 300 K, did not exhibit significant magnetic properties. An increase in the synthesis time leads to the formation of magnetite nanoparticles, which is confirmed by XRD analysis (Figure 2a). An increase in the content of the Fe_3_O_4_ phase leads to a growth in M_s_ values up to 46.83 ± 0.29 emu/g (synthesis time 12 h). This is confirmed by the data on the phase composition, which are presented in Table 1. The saturation magnetization is much lower than the M_s_ value of a magnetite polycrystal, which is 92 emu/g [50]. This is due to the low content of the magnetic phase and the low-dimensional state of Fe_3_O_4_ crystallites (Figure 5). Another reason for the decrease in magnetic properties both at the beginning and at the end of magnetite synthesis is that the α-FeOOH particles surrounding the Fe_3_O_4_ particles reduce the magnetic dipole interactions between neighboring magnetic particles.

A further increase in the synthesis time of magnetite particles leads to a decrease in the M_s_ value to 1.78 ± 0.04 emu/g (the synthesis time is 24 h). This is explained by the transformation of Fe_3_O_4_ into the antiferromagnet α-FeOOH at a Curie temperature of 393 K. Despite α-FeOOH being antiferromagnetic, it has a nonzero magnetic moment due to incomplete compensation of the magnetic moments of the sublattices when in the form of nanoparticles.

While hysteresis losses are practically absent, the coercive force increases with the duration of the synthesis. The higher H_c_ values as compared to pure magnetite [51] can be explained in terms of the presence of the α-FeOOH phase in the samples. Due to the canting of the moments in the magnetic structure of goethite, four sublattices can be distinguished instead of two. As a result, the coercivity of pure goethite can reach high values, which is possibly attributable to permanent magnetism [52]. It should be mentioned that M_s_ values in the case of ANPs of Fe_3_O_4_ obtained by the other research groups differ from both the analogical isotropic nanoparticles and the bulk material [53]. For instance, M_s_ of Fe_3_O_4_ ANPs is 84 [54], 54 [32], 28 [35], and 17 [9] emu/g (more values presented in Table 5). Such an inhomogeneity in the values of the M_s_ is associated with high shape anisotropy of ANPs which prevents them from magnetization in directions other than along their easy magnetic axes [29], with the increase in surface spin canting effect depending on the particle size [53], and with a presence of nonmagnetic side phases [23]. The latter factor has a greater effect on the M_s_ in the case of the synthesis of ANPs obtained by the co-precipitation method. Thus, to reliably confirm the structure and phase composition in order to explain the magnetization characteristics of ANPs, it is necessary to use a combination of XRD, Raman, HRTEM, and pulse magnetometry or vibrating-sample magnetometry (VSM).

**Figure 8:**
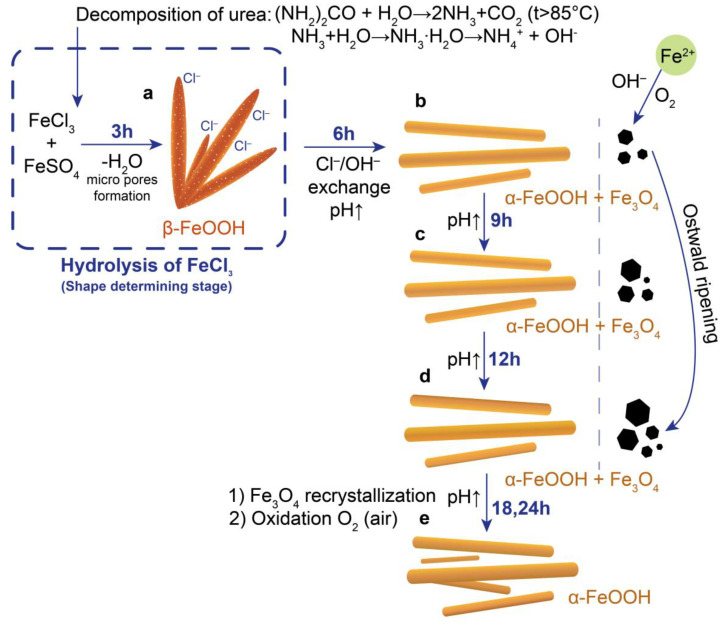
Pathway of iron derivatives transformations during co-precipitation of Fe^2+^ and Fe^3+^ salts via decomposition of urea after 3 (**a**), 6 (**b**), 9 (**c**), 12 (**d**), and 18–24 h (**e**) of the synthesis.

Based on obtained results and the mechanism suggested by Lian et al. [27], the possible pathway of reaction during ANPs formation was proposed. While the observed chemical processes during ANP synthesis are in general agreement with the reactions described in the Materials and Methods section, some differences are observed. A scheme of the proceeding stages is presented in Figure 8. Since magnetite ANPs is obtained by the co-precipitation of iron salt in two oxidation states, the solubility of formed hydroxides (oxyhydroxides) should be taken into account for the determination of reaction routes. Since the solubility product of Fe(OH)_3_ (K_sp_(Fe(OH)_3_) = 2.79∙10^−39^) is much smaller than that of Fe(OH)_2_ (K_sp_(Fe(OH)_2_) = 4.87∙10^−17^) [55], Fe(OH)_3_ is the first to be precipitated during the reaction. As one of the condensed aqua-hydroxo complexes, Fe(OH)_3_ is known not to exist in solution in such a form [56].

At first, hydrolysis of FeCl_3_ is accompanied by the formation of β-FeOOH nanoellipsoids (including dehydration of iron hydroxide) that act as a template for the further synthesis of ANPs (Figure 2a and Figure 3a). Thus, this process can be considered as a shape-determining stage. Predominant formation of β-FeOOH at the earliest stages is due to the stabilization of its structure by Cl^−^ ions incorporating into the tunnels located in it structure (Figure 8a) [57]. The initial concentration of FeCl_3_ affects the size of β-FeOOH nanoellipsoids [58]. For this reason, it should be taken into account when synthesizing ANPs of a specified shape and size.

During synthesis at 95 °C, a gradual decomposition of urea occurs to generate OH^−^ ions. With increasing synthesis time and pH of the solution, Cl^−^ ions are replaced by OH^−^ ions to transform β-FeOOH into α-FeOOH (Figure 8b). While the adsorption of Fe^2+^ ions onto the α-FeOOH (Fe^3+^ source) surface at the next stage proceeds according to a mechanism suggested by Lian et al. [27], this does not occur in our synthesis. To explain this regularity, we should consider two possible reaction pathways in which Fe^2+^ ions can participate. On the one hand, Fe^2+^ ions can adsorb on the surface of α-FeOOH ANPs and then transform to Fe_3_O_4_ ANPs during further precipitation reaction (Figure 8). On the other hand, Fe^2+^ ions can be oxidized by air oxygen (the reaction system is not isolated) and form isotropic submicron nanoparticles of Fe_3_O_4_ (Figure 8b–d). However, the latter does not lead to the formation of anisotropic morphology in the case of Fe_3_O_4_ due to its characteristic cubic crystal structure and different rate of Fe^2+^ and Fe^3+^ deposition. The probability of one of the occurring processes will be determined by their thermodynamic and kinetic regularities. The kinetics of Fe^2+^ oxidation was demonstrated by Morgan and Lahav [59]. They noticed a high rate of the oxidation process, which can be thermodynamically and kinetically enhanced by surface hydroxyl groups of α-FeOOH. Later, Chen and Thompson found that the rate of Fe^2+^oxidation increases significantly in the presence of α-FeOOH (4 h vs. a few minutes), which catalyzes the process [60]. These statements, which are in good agreement with our results, explain the formation of isotropic submicron Fe_3_O_4_ particles. It is important to note that, although γ-Fe_2_O_3_ can also form, its presence is difficult to determine by ex situ methods [19]. The formation of α-Fe_2_O_3_ can be observed due to its greater thermodynamic stability as demonstrated by the HRTEM method. A further increase in the synthesis time (up to 12 h) is accompanied by the oxidation of Fe_3_O_4_ to α-FeOOH ANPs at high pH due to the presence of air oxygen (Figure 8b–d) [61]. After 24 h of synthesis, almost all of the Fe_3_O_4_ converts to α-FeOOH (Figure 8e) as confirmed by XRD and Raman spectroscopy results. The dependence of M_s_ on synthesis time is also in agreement with proposed reaction pathway. After 12 h of synthesis, M_s_ decreases to an almost zero value to support a very low quantity of Fe_3_O_4_ due to its transformation to α-FeOOH. Thus, while the co-precipitation method is not suitable for the synthesis of pure Fe_3_O_4_ ANPs, it can be used as a method of synthesis of α- and β-FeOOH with given morphology.

Along with the features of the reactions occurring during the synthesis of ANPs of iron derivatives, the set of research methods and the sequence of their use are of great importance. The known sets of analytical methods used in the study of ANPs are summarized in Table 5. Although the XPS method cannot be used to distinguish mixture of iron phases due to the close values of binding energies, it is the most informative approach for the analysis of nanoparticles with a modified surface. For this reason, XPS is not included in Table 5. A review of the methods used for the study of iron oxides and oxyhydroxides ANPs (Table 5) shows that the most commonly used research methods are XRD, HRTEM, and VSM, which are used to characterize phase composition, morphology (crystal structure), and magnetic properties, respectively. From the point of view of phase composition, determination of the maghemite (γ-Fe_2_O_3_) phase by XRD is difficult due to the similarity of its reflection (and crystal structure) to Fe_3_O_4_. Thus, another method or combination of methods is required. For instance, Raman spectroscopy, which represents a powerful method for determining a γ-Fe_2_O_3_ phase which demonstrates a characteristic band at 700 cm^−1^ of Raman shift [62]. Here, it is necessary to avoid high laser power that can affects the real phase composition [63]. Although infrared spectroscopy is a less informative method for iron oxygen-containing compounds, it can be useful for analyzing modified iron oxides (hydroxides) [64] and their active sites by adsorption of specific probe molecules in the case of catalytic application [65]. The morphology and shape of iron-based ANPs are studied by SEM and TEM (HRTEM). While analysis of SEM and HRTEM images can be used to demonstrate the shape and size of synthesized ANPs or isotropic nanoparticles, a comprehensive analysis of ANPs should include evaluation of interplanar spacings; however, for iron- and oxygen-containing compounds, these may have similar values. Thus, it becomes necessary to measure several values of interplanar spacings and angles between definite crystallographic planes [66]. In the case of magnetic iron oxide ANPs, M_s_ is a basic parameter that should be measured to determine their magnetic characteristics. Saturation magnetization mainly depends on phase composition, particle size, and the presence of organic or polymer surface modifiers [63]. However, M_s_ is usually measured both to confirm the purity of the magnetic phase and to evaluate its potential as a magnetic component of magnetoactive materials for different application areas.

**Figure 9:**
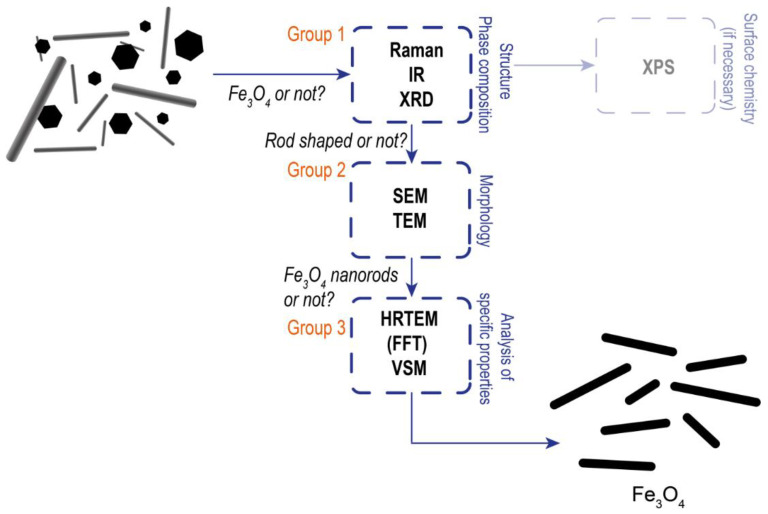
Methodological scheme of analysis of ANPs based on iron oxide or oxyhydroxide (an example for Fe_3_O_4_ nanorods).

A methodological scheme for the analysis of iron oxide and oxyhydroxide ANPs based on our results and those of other studies is presented in Figure 9. The first group of methods is aimed at establishing the structure, phase, and functional composition of studied nanoparticles. First, it is necessary to confirm by XRD that the studied sample is a monophasic compound containing exactly the target phase. In addition to data on the phase composition, Raman and Infrared spectra should be registered to identify the presence of all iron oxides and oxyhydroxides ANPs that cannot be reliably determined by XRD (as an example, γ-Fe_2_O_3_). Moreover, IR spectroscopy and XPS are useful for analysis of the modified surface of ANPs. The second group of methods, including SEM and TEM (HRTEM), are used to determine the morphology of the studied iron-based nanoparticles. As well as being used to confirm the anisotropic shape and form of the synthesized nanoparticles, these methods are used to calculate the size distribution and evaluate the tendency to agglomeration. The third group of methods is aimed at confirming the phase composition and crystal structure of ANPs, as well as studying their magnetic properties. The use of these methods, along with traditional methods for studying the structure, phase, and functional composition (methods of group 1), is due to the rather similar properties of various iron oxides and oxyhydroxides, which complicates the reliable assessment of the properties of studied iron based ANPs. Assignment of ANPs to a certain phase should be carried out based on detailed FFT analysis of several interplanar spacings corresponding to a definite set of crystallographic planes and the angles between them. In the case of magnetic iron oxide ANPs, their magnetic properties should be determined, mainly in terms of M_s_. In general, the analysis of hysteresis curves is used to estimate the value of the M_s_, coercivity, residual magnetization, as well as to determine the magnetic behavior of nanoparticles (e.g., paramagnetic, ferromagnetic, ferrimagnetic). Thus, a proposed methodological scheme is aimed at the standardization of research methods for obtaining the most reliable data on the structure, phase composition, and other important properties of iron oxides and oxyhydroxides ANPs. This methodological scheme will be also useful not only for iron-based ANPs, but also for isotropic ones, as obtained by different synthesis procedures.

**Table 5:** Characterization methods for a study of iron oxide and oxyhyroxide ANPs presented in some researches.

Type of ANPs	Methods of Analysis						
	Structure, Phase Composition, and Functional Groups			Morphology		Magnetic Properties	Ref.
	XRD	Raman	IR	SEM	TEM [FFT] ^a^	VSM M_s_ [Coercivity]	
β-FeOOH (a), Fe_3_O_4_ (b) nanorods	(a) β-FeOOH phase (b) Fe_3_O_4_ phase	**–**	**–**	**–**	(a) D ^b^ = 10–50 nm L ^c^ = 50–400 nm (b) D = 15 nm L = 45, 400 nm [(311) plane, d ^d^ = 0.2 nm]	(b) 78 emu/g [38 Oe for AR ^e^ = 4.5, 334 Oe for AR = 10]	[67]
Fe_3_O_4_ nanowires	Fe_3_O_4_ phase	**–**	**–**	**–**	D = 20 nm L = 800 nm [(111) plane, d = 0.48 nm]	35.2 emu/g [not studied]	[29]
Fe_3_O_4_, spherical (a), cubic (b), rod shaped (c)	(a) Fe_3_O_4_ phase (b) Fe_3_O_4_ phase (c) Fe_3_O_4_ phase	**–**	**–**	(a) L = 25 nm (b) L = 63 nm (c) D = 12 nm L = 60–120 nm	**–**	(a) 60.7 emu/g (b) 60.4 emu/g (c) 35.4 emu/g [not studied]	[53]
Fe_3_O_4_ nanobelts	Fe_3_O_4_ phase (XRD pattern contains reflections of side phase)	**–**	**–**	**–**	D = 70–90 nm L = 10–15 μm	54 emu/g [not studied]	[32]
Fe_3_O_4_ nanorods	Fe_3_O_4_ phase	**–**	**–**	Particles size is not discussed	D = 10 nm L = 150 nm [not studied]	28.7 emu/g [not studied]	[33]
Fe_3_O_4_ nanorods	Fe_3_O_4_ phase	**–**	597 cm^−1^ (Fe–O vibrations in Fe_3_O_4_) ^f^	**–**	D = 8–64 nm L = 58–250 nm [(220) plane, d = 0.29 nm]	71.3 emu/g [not studied]	[64]
Fe_3_O_4_ nanorods	Fe_3_O_4_ phase	672 cm^−1^ (A_1g_ mode of Fe_3_O_4_) bands of other iron oxides and oxyhydroxydes (γ-Fe_2_O_3_, α-FeOOH)	**–**	**–**	D = 10 nm L = 150 nm [(220) plane, d = 0.296 nm; (200) plane, d = 0.42 nm]	**–**	[66]
γ-Fe_2_O_3_ (a), α-FeOOH (b), γ-FeOOH (spherical, rod shaped)	Individual γ-Fe_2_O_3_, α-FeOOH, γ-FeOOH phases of their mixture ^g^	**–**	449 and 632 cm^−1^ Fe–O stretching 583 cm^−1^ typical band of Fe_2_O_3_	**–**	Particles size is not discussed [(220) plane, d = 0.296 nm; (200) plane, d = 0.42 nm]	54 emu/g [not studied]	[68]
Fe_3_O_4_, rod shaped (a), cubic (b), spherical (c)	(a) Fe_3_O_4_ phase (XRD contains side reflections) (b) Fe_3_O_4_ phase (c) Fe_3_O_4_ phase	(a–c) 300, 540, and 670 cm^−1^ (vibration modes of Fe_3_O_4_) 350, 500, and 700 cm^−1^ (vibration modes of γ-Fe_2_O_3_)	(a) 896, 796, and 628 cm^−1^ (characteristic bands of α-FeOOH) (b) and (c) 580 cm^−1^ (Fe–O vibrations in Fe_3_O_4_)	**–**	(a) D and L of rods are not discussed (b) L = 30 nm (c) L = 13 nm [not studied]	(a) 52 emu/g [48 Oe] (b) 81 emu/g [61 Oe] (c) 60 emu/g [0 Oe]	[69]
α-FeOOH (a), α-Fe_2_O_3_ (b), Fe_3_O_4_ (c) nanorods	(a) α-FeOOH phase (b) α-Fe_2_O_3_ phase (c) Fe_3_O_4_ phase	**–**	**–**	(a) D = 70 nm L = 500 nm (b) D = 70 nm L = 120–200 nm (c) D = 70 nm L = 500 nm	Particles size is not discussed (a) [(110) plane, d = 0.42 nm] (b) [(012) plane, d = 0.368 nm; (110) plane, d = 0.251 nm] (c) [(311) plane, d = 0.25 nm; (400) plane, d = 0.2 nm]	**–**	[70]
β-FeOOH (a), Fe_3_O_4_ (b) nanorods	(a) β-FeOOH phase (b) Fe_3_O_4_ phase	**–**	(a) 556, 614, 695 and 825 cm^−1^ (Fe–O vibrational modes of β-FeOOH) (b) 569 cm^−1^ (Fe–O vibrational mode of Fe_3_O_4_)	**–**	(a) D = 3–12 nm L = 25–70 nm (b) D = 3–12 nm L = 30, 40, 50, 60, 70 nm [not studied]	(b) 50–66 emu/g for 30–70 nm ANPs [not studied]	[71]
β-FeOOH (a), Fe_3_O_4_ (b) (spherical, ellipsoids, hollow ellipsoids)	(a) β-FeOOH phase (b) Fe_3_O_4_ phase	**–**	**–**	**–**	(a) D = 38 nm L = 172 nm (b) D = 38 nm (spheres); D = 38 nm L = 172 nm (ellipsoids); D = 38 nm L = 172 nm (hollow ellipsoids) [not studied]	(b) 84.2 emu/g [5.0 Oe] (spheres); 65.6 emu/g [6.5 Oe] (ellipsoids); 53.0 emu/g [37.8 Oe] (hollow ellipsoids)	[72]

^a^ Data of the corresponding method presented in square brackets; ^b^ D—diameter of ANPs; ^c^ L—length of ANPs; ^d^ d—interplanar spacing; ^e^ AR—aspect ratio; ^f^ IR spectra also include bands of Fe_3_O_4_ modifiers; ^g^ Phase composition depends on initial Fe(NO_3_)_3_ concentration.

## 4. Conclusions

The synthesis of iron oxide and oxyhydroxide ANPs was carried out by co-precipitation of Fe^2+^ and Fe^3+^ salt in the presence of urea. By varying the synthesis time, changes of their morphology, phase, and chemical composition, as well as their magnetic properties, were studied by SEM, XRD, Raman spectroscopy, XPS, HRTEM, and pulse magnetometry. XRD and Raman spectroscopy results demonstrate that β-FeOOH is formed at the early stages of the synthesis. The formation of the Fe_3_O_4_ phase occurs until 12 h of the synthesis and reaches a maximum Fe_3_O_4_ phase content of 47.4%. The subsequent increase of time leads to predominant formation of α-FeOOH phase due to oxidation of Fe_3_O_4_ by air oxygen. Such observations are in good agreement with M_s_ values which reach a maximum (46.83 emu/g) after 12 h of the synthesis. The presence of both isotropic and anisotropic particles after 12 h is confirmed by SEM and HRTEM. The latter in combination with FFT analysis allows determining phase composition via measurements of interplanar spacings and angles between specific crystallographic planes.

Based on the obtained experimental data, refined reaction pathways of the formation of iron oxides and oxyhydroxides ANPs are proposed. The hydrolysis of FeCl_3_ occurring at the beginning of the process (3 h) leads to the formation of β-FeOOH nanoellipsoids due to the stabilization of its structure by Cl^−^ ions. This stage can be considered as shape-determining due to the formation of rod-shaped structures depending on the initial concentration of FeCl_3_. Then, β-FeOOH to α-FeOOH transformation takes place because of the exchange of Cl^−^ by OH^−^ ions forming via urea decomposition. At increased synthesis time and OH^−^ ion concentration, along with rod-shaped α-FeOOH nanoparticles, isotropic Fe_3_O_4_ submicron particles are formed during Fe^2+^ oxidation and Ostwald ripening process. Compared to the adsorption of Fe^2+^ ions on the surface of rod-shaped α-FeOOH, which should lead to the formation of Fe_3_O_4_ nanorods, these tend to favor kinetically rapid oxidation accompanied by the formation of submicron isotropic Fe_3_O_4_ particles. The isotropic shape of Fe_3_O_4_ particles connected with their cubic crystal structure and the difference in Fe^2+^ and Fe^3+^ deposition are factors that hinder the formation of ANPs. Along with a description of the chemistry of the process, a methodology for analyzing ANPs based on iron oxide or oxyhydroxide is proposed. Such a methodological scheme will be useful for carrying out a detailed analysis of the chemistry, phase composition, and structure of iron oxides and oxyhydroxides ANPs.

## Figures and Tables

**Table 1:** Phase composition of the samples at different time of the synthesis.

Synthesis Time, h	Phase Composition, wt.%		
	Fe_3_O_4_	β-FeOOH	α-FeOOH
3	0	100	0
6	35.8	0	64.2
9	46.7	0	53.3
12	47.4	0	52.6
18	6.1	0	93.9
24	1.9	0	98.1

**Table 2:** Position of Fe 2p peaks and Fe/O ratio in the samples at different time of synthesis.

Time, h	Fe 2p_3/2_	Fe 2p_1/2_	Fe/O
3	710.9	724.6	0.29
6	711.0	724.6	0.37
9	710.1	723.8	0.37
12	710.9	724.7	0.38
18	711.4	725.1	0.36
24	711.3	724.9	0.37

**Table 3:** Interplanar spacings of iron oxide and oxyhydroxide forming during ANPs synthesis.

Fe_3_O_4_ [42]		α-FeOOH [41,43]		α-Fe_2_O_3_ [44]	
d, Å	(hkℓ)	d, Å	(hkℓ)	d, Å	(hkℓ)
4.81	(111)	4.97	(020)	3.7	(012)
2.96	(220)	2.58	(021)	2.7	(104)
2.52	(311)	4.20	(110)	2.52	(110)
2.41	(222)	2.58	(101)	2.1	(202)
2.09	(400)	2.45	(111)		
1.71	(422)	2.25	(121)		
1.61	(511)	1.71	(212)		
1.28	(533)	2.52	(040)		

**Table 4:** Magnetic properties of obtained iron oxide and oxyhydroxide nanoparticles.

Time, h	M_s_ ^a^, emu/g	M_r_ ^b^, emu/g	H_c_ ^c^, Oe	Fe_3_O_4_ Content ^d^, %
3	0.3 ± 0.01	0.006	0	0
6	30.79 ± 0.18	2.56 ± 0.05	75 ± 2.25	35.8
9	43.82 ± 0.06	2.63 ± 0.05	90 ± 2.70	46.7
12	46.83 ± 0.29	2.71 ± 0.05	103 ± 3.09	47.4
18	7.78 ± 0.21	0.87 ± 0.02	118 ± 3.54	6.1
24	1.78 ± 0.04	0.15 ± 0.01	120 ± 3.60	1.9

^a^ M_s_—saturation magnetization; ^b^ M_r_—remanent magnetization; ^c^ H_c_—coercivity; ^d^ Calculated based on XRD data using a Match! Software.

## Data Availability

The data that support the findings of this study are available from the corresponding author upon reasonable request.
